# Invasive African clawed frogs in California: A reservoir for or predator against the chytrid fungus?

**DOI:** 10.1371/journal.pone.0191537

**Published:** 2018-02-14

**Authors:** Emily A. Wilson, Cheryl J. Briggs, Tom L. Dudley

**Affiliations:** 1 Marine Science Institute, University of California Santa Barbara, Santa Barbara, California, United States of America; 2 Department of Ecology, Evolution, and Marine Biology, University of California Santa Barbara, Santa Barbara, California, United States of America; Stockholm University, SWEDEN

## Abstract

Amphibian species are experiencing population declines due to infection by the fungal pathogen, *Batrachochytrium dendrobatidis* (Bd). The African clawed frog (*Xenopus laevis*), an asymptomatic carrier of Bd, has been implicated in the spread of this pathogen through global trade and established invasive populations on several continents. However, research has not explored the relationships of both life stages of this amphibian with Bd. While the post-metamorphic individuals may act as a reservoir, spreading the infection to susceptible species, the filter-feeding larvae may consume the motile Bd zoospores from the water column, potentially reducing pathogen abundance and thus the likelihood of infection. We explore these contrasting processes by assessing Bd prevalence and infection intensities in field populations of post-metamorphic individuals, and performing laboratory experiments to determine if larval *X*. *laevis* preyed upon Bd zoospores. The water flea, *Daphnia magna*, was included in the Bd consumption trials to compare consumption rates and to explore whether intraguild predation between the larval *X*. *laevis* and *Daphnia* may occur, potentially interfering with control of Bd zoospores by *Daphnia*. Field surveys of three *X*. *laevis* populations in southern California, in which 70 post-metamorphic individuals were tested for Bd, found 10% infection prevalence. All infected individuals had very low infection loads (all Bd loads were below 5 zoospore equivalents). Laboratory experiments found that larval *X*. *laevis* consume Bd zoospores and therefore may reduce Bd abundance and transmission between amphibians. However, metamorphic and juvenile *X*. *laevis* exhibited intraguild predation by consuming *Daphnia*, which also prey upon Bd zoospores. The results suggest that *X laevis* is not a large reservoir for Bd and its larval stage may offer some reduction of Bd transmission through direct predation.

## Introduction

The amphibian pathogen, *Batrachochytrium dendrobatidis* (Bd), is responsible for population declines and extinctions of many amphibian species worldwide [1, 2]. Understanding the role of Bd in amphibian declines is complicated by its uneven impacts across species. While some species experience rapid mortality when infected, others are asymptomatic carriers that suffer no negative effects from the infection [3]. Species vary in their potential to transmit the pathogen to other organisms, in that some species or life stages can act as a reservoir, harboring the pathogen that subsequently infect other amphibians [4, 5], while other species may exhibit behaviors that reduce the abundances or infective potential of the pathogen, which reduces transmission potential [6, 7].

Reservoir species are infected carriers, often displaying few symptoms of infection [8]. These carrier species can be detrimental to susceptible species by facilitating pathogen retention in an environment following the extirpation of susceptible amphibian species. As a result, the ability of the populations to rebound following an initial pathogen driven die-off may become more difficult for susceptible species [5]. Many species have been found to carry Bd and may act as reservoirs, spreading the pathogen to susceptible amphibians. Two invasive amphibian species, the African clawed frog (*Xenopus laevis*) and American bullfrog (*Lithobates catesbeianus*), are likely Bd reservoirs and are often implicated as spreading the pathogen globally [9, 10, 11]. North American crayfish species (*Procambarus spp*. and *Orconectes virilis*) can also harbor and transmit Bd infection and given their widespread invasion into waterways, could be an important reservoir of Bd [4]. Native amphibian species can also act as fungal reservoirs. Amphibians such as the Pacific treefrog (*Pseudacris regilla*) can sustain an infection with little evidence of disease but may spread the pathogen as they move across a landscape [5].

Conversely, within the aquatic environment, consumers capable of feeding on infective stages can potentially function as biological control agents suppressing the pathogen abundance. Bd transmission occurs through a motile zoospore stage that swims through the water to infect a new host or re-infect the current host. Zooplankton, such as *Daphnia* [6, 7, 12, 13] and ciliates [14], consume the motile zoospore stage of Bd from the water column. The reduction of Bd zoospores may lead to reduced transmission rates to amphibians in the water [7, 13, 14].

*X*. *laevis* is unique among potential reservoir species because it may function as both a reservoir and a predator of the pathogen, depending on its life stage. Adult *X*. *laevis* are asymptomatic carriers of Bd [15]. The species is fully aquatic and could expose native amphibians to the infectious stage of Bd if they share water sources. *X*. *laevis* larvae, in contrast, do not become infected with Bd, but as obligate filter feeders, the larval stage of this species has the potential to function as a Bd predator by consuming Bd zoospores from the water column.

To understand the potential of adult *X*. *laevis* as a reservoir in a field population, it is necessary to consider both the proportion individuals that are infected (i.e. the Bd ‘prevalence’), and the intensity of infection per individual (the infection ‘load’). The higher the infection intensity an individual has, the larger the zoospore output and thus the higher potential for transmission [16]. Bd infection prevalence and loads vary regionally in *X*. *laevis* populations (Table 1). In its native range in sub-Saharan Africa, *X*. *laevis* infection prevalence range from 0.25% [9, 17] to 25.2% [18]. In its introduced range, *X*. *laevis* in the UK displayed the greatest variation in Bd infection prevalence (from 0% and 83.6%), with correspondingly large variation in infection loads, depending on location and season [19]. In Chile, only three out of ten sites with *X*. *laevis* were Bd positive, all with low infection loads; however, the overall infection prevalence across all sampled individuals was 24% [20]. In France, a population was surveyed for Bd but none of the specimens was positive [21] and in Japan a population was estimated to have an infection prevalence of 13% [22]. In California, *X*. *laevis* infection levels and prevalence have been estimated only from preserved specimens. One study of museum specimens estimated prevalence at 13% and all infection loads were less than one genetic equivalent (GE) [17]. A separate analysis found that previously collected specimens estimated infection prevalence at 4% in California [18].

**Table 1:** Previously published results of Bd infection found on *X*. *laevis*.

Author	Region	Specimen Type	Detection Method	Number Specimens	Prevalence	Load (GE)
Weldon et al. 2005	South Africa	Live Capture	Histology—toe webbing	365	25.2%	N/A
Weldon et al. 2004	Africa	Preserved	Histology—toe webbing	583	2.6%	N/A
Soto-Azat et al. 2010	Africa	Preserved	qPCR—swab*	249	1.2%	≤ 10.3
Vredenberg et al. 2013	Africa	Preserved	qPCR—swab	122	0.25%	≤ 2
Solis et al. 2010	Chile	Live Capture	qPCR—toe clip	58	24%	≤ 10
Tinsley et al. 2015	UK	Live Capture	qPCR—swab	221	0–83.6%	range^†^
Ouellet et al. 2012	France	Live Capture	Histology—toe clip	89	0%	N/A
Goka et al. 2009	Japan	Live Capture	PCR—swab	168	13%	N/A
Vredenberg et al. 2013	California, USA	Preserved	qPCR—swab	23	13%	≤ 1
Weldon et al. 2005	California, USA	Preserved	Histology—toe webbing	102	4%	N/A

**S**pecimens from African are within their native ranges while species from other regions are introduced populations.

*Included larval *X*. *laevis*.

^†^Bd prevalence and load varied widely based on site and season.

With such variation in *X*. *laevis* infection prevalence and loads globally, it is difficult to say with certainty what infection levels *X*. *laevis* populations will have in a particular region and season. There appears to be potential for invasive *X*. *laevis* populations to act as a reservoir for Bd, capable of driving a Bd outbreak in an area where individuals are harboring high loads or have high prevalence of infection [19, 20]. There are also populations of *X*. *laevis* that either do not harbor Bd or have low infection prevalence and loads [18, 19, 21]. Many of these specimens were collected decades ago, adding additional uncertainty concerning the current status of *X*. *laevis* infection so an update evaluation of *X*. *laevis* infection levels is needed to determine is current status as a potential reservoir in southern California.

The potential for larval *X*. *laevis* to act as potential predators of Bd has not yet been investigated. Zoospores average of 3–5 μm in diameter [23] and larval *X*. *laevis* are capable of removing particles from 0.2 μm to over 200 μm from the water [24]. Larval *X*. *laevis* cannot be a source of Bd because the zoospores infect only the keratinized structures found in the skin of post-metamorphic amphibians and the mouthparts of larval anurans [25]. As filter feeding specialists, *X*. *laevis* larvae lack keratinized tooth-like mouthparts used for grazing [26] and therefore cannot harbor a Bd infection. Only when the *X*. *laevis* metamorphose do they produce keratinized structures in their skin that are susceptible to infection.

Although larval *X*. *laevis* have the potential to be effective at removing Bd from the water column, they may also interfere with Bd control by zooplankton species that also consume Bd. Zooplankton are a fraction the size of *X*. *laevis* larvae so their filtration rates cannot rival a *X*. *laevis* larva. *X*. *laevis* may also have a lower particle threshold than zooplankton, making them capable of feeding at lower particle concentrations [24]. Zooplankton could compensate for their smaller size, however, by their potential to occur at higher densities than *X*. *laevis* in aquatic communities. To evaluate how effective *X*. *laevis* larvae are we compared their Bd zoospore consumption rate to that of a zooplankton species, *Daphnia magna*.

The impact of larval *X*. *laevis* as a predator suppressing Bd zoospore abundance may be offset by the potential of *X*. *laevis* larvae and juveniles to act as intraguild predators on native zooplankton that also feed on Bd. Zooplankton such as *Daphnia* have been suggested as a potential controls for Bd [6, 14] so any significant loss in natural zooplankton populations could negate positive effects of predation by *X*. *laevis* larvae on Bd. Larval *X*. *laevis* are known to filter small prey particles out of the water column and transition to larger zooplankton as they metamorphose and develop into juveniles [27]. It is therefore necessary to determine the developmental stage at which *X*. *laevis* could become an intraguild predator and start consuming the relatively large zooplankton such as *D*. *magna*.

This study explores the potential for *X*. *laevis* to act as a reservoir for, and/or a predator against, Bd in southern California. To test whether *X*. *laevis* are a reservoir for Bd, post-metamorphic *X*. *laevis* wild populations in southern California were surveyed and tested for Bd infection to determine prevalence and load of infection. To test whether larval *X*. *laevis* prey on Bd, laboratory experiments were performed determine if larval *X*. *laevis* consume Bd zoospores from the water column and if those consumption rates are comparable to those of *Daphnia*. Laboratory predation trial with *X*. *laevis* larvae and juveniles were performed to determine at what developmental stage *X*. *laevis* become capable of consuming large zooplankton that also feed on Bd zoospores.

## Materials and methods

All necessary permits were obtained from the described study, which complied with all relevant regulations. Research was performed following the UCSB Institutional Animal Care and Use Committee protocols approved for this project (735 & 865). All *X*. *laevis* were handled as little as possible to minimize stress and humanely euthanized by sitting the animals in a bath of buffered pharmaceutical grade tricaine methanesulfonate for no less than one hour. Fieldwork was performed with approval from the California Department of Fish and Wildlife (Scientific Collection Permit 12279).

### Wild *X*. *laevis* Bd infection levels

Individual *X*. *laevis* were captured in the field and evaluated for Bd infection prevalence and load. The *X*. *laevis* were collected from 3 sites: an isolated pond on Hedrick Ranch Nature Area, adjacent to the Santa Clara River, Ventura County; isolated pools on Piru Creek, Ventura County; and Murray Canyon Creek, San Diego County. Any other amphibian species present at the sites were included in the study when found but other species were not specifically targeted. Individuals were captured by funnel minnow trap, seine or dipnet. Individuals were handled with clean gloves and their ventral surfaces were swabbed with a sterile cotton-tip swab following the protocol of Hyatt et al. [28] to collect Bd cells for genetic detection. Swabs were either field dried and stored at room temperature or if not dried, stored at -4°C.

Swabs were processed in triplicate using a quantitative PCR assay following the protocol of Boyle et al. [29] with Life Technologies Taqman Universal Master Mix or Bioline Sensifast Master Mix. Bd amplification standards of 0.1, 1, 10, 100, and 1,000 zoospore equivalents, isolated from 60 Lakes Basin, Kings Canyon National Park in 2009, were included in each assay to quantify the amount of Bd on each swab. An individual was considered infected if a single replicate was positive for Bd. An individual’s Bd infection load was calculated by averaging all positive quantitative PCR results from the individual’s three replicates.

### Larval *X*. *laevis* & *Daphnia* consumption of Bd

A laboratory experiment was performed to determine if larval *X*. *laevis* consume Bd zoospores, and if so, how their consumption rates compare to adult *Daphnia*. The larval *X*. *laevis* were purchased commercially (Nasco_®_), fed Nasco Frog Brittle powder *ad libitum*, and then fasted 24 hours prior to the experiment. The *D*. *magna* were purchased commercially (Ward’s Scientific), fed yeast powder *ad libitum*, and then fasted 24 hours prior to the experiment. *Daphnia magna*, hereafter referred to as ‘*Daphnia*,’ were chosen as representatives of zooplankton because they are common research zooplankton that can be purchased from a commercial supplier and have been used in previous work with Bd [6].

The experiment was performed in 400 mL plastic cups filled with 120 mL of purified bottled water. One *X*. *laevis* larva or three adult *Daphnia* were placed in one of the six treatments: Bd present with a live *X*. *laevis* larva (n = 16); Bd present with a dead *X*. *laevis* larva (n = 8); Bd present with live *Daphnia* (n = 8); Bd present with dead *Daphnia* (n = 4); Bd absent with a live *X*. *laevis* larva (n = 5); Bd absent with live *Daphnia* (n = 3). Treatments with live *X*. *laevis* had a greater number of replicates than the live *Daphnia* to capture variation in within the size ranges of the larvae.

The treatments that exposed dead *X*. *laevis* or dead *Daphnia* to Bd were included in the experiment to distinguish actively consumed Bd zoospores from any zoospores that might inadvertently swim in the mouth of a *X*. *laevis* or attach to the carapace of the *Daphnia*. The treatments with dead potential consumers or without Bd used a smaller number of replicates because they were considered negative and contamination controls rather than behavioral experiments. The larval *X*. *laevis* were euthanized in a buffered MS222 solution (5g/L) for one hour and the adult *Daphnia* were euthanized in 70% ethanol. The euthanized animals were then rinsed twice with fresh water before placement into the experiment.

The Bd was cultured in the laboratory from the CJB7 isolate collected from Sixty Lake Basin in Kings Canyon National Park, California. The concentration of Bd zoospores was counted using a hemocytometer. Each replicate of the treatments containing Bd was inoculated with 442,000 zoospores.

The experiment ran for 4.5 hours, after which all animals were removed from the treatments and rinsed thoroughly with fresh water. Live *X*. *laevis* and *Daphnia* were immediately euthanized with MS222 or ethanol, respectively. All animals were then preserved in ethanol. The gut of each *X*. *laevis* was dissected from esophagus to vent and cut into pieces. The *Daphnia* in each treatment were pulverized with a 1.5mL vial pestle in preparation for DNA extraction. DNA extraction was performed using the Qiagen DNeasy Blood & Tissue Kit and protocol with the exception that the tissues were incubated overnight in the lysis step to facilitate complete tissue breakdown. Each sample was analyzed in duplicate using the same quantitative PCR protocol described in the “Wild *X*. *laevis* Bd infection levels” section; the two runs for each sample were averaged.

### Larval *X*. *laevis* predation on *Daphnia*

A second laboratory experiment was performed to measure *X*. *laevis* predation on *Daphnia*. The animals were procured from the same commercial suppliers and had the same diet as the animals used in the previous experiment, except that the juvenile *X*. *laevis* were fed small pellets of Nasco Frog Brittle. The zooplankton, *Daphnia*, were divided into three separate classes based on size measured from the crown of the head to the base of the spine: neonates (≤ 1 mm), juveniles (< 1 to 2.25 mm); adults (≥ 2.25 mm) [30, 31].

Five *Daphnia* of the same size class were placed in 400 mL cups filled with 200 mL of purified bottle water. A single larval (Gosner stages 26–42), metamorphic (Gosner stages 45–46), or juvenile (SVL 24–30 mm) [32] *X*. *laevis* was fasted for 24 hours prior to the experiment and placed in each cup with the *Daphnia*. The experiment was performed in the following factorial design: a larval *X*. *laevis* with 5 individual Daphnia (n = 4 replicates of each *Daphnia* size class; 12 cups total); a metamorphic *X*. *laevis* with 5 individual *Daphnia* (n = 2 replicates of each *Daphnia* size class; 6 cups total); and a juvenile *X*. *laevis* with 5 individual *Daphnia* (n = 2 replicates of each *Daphnia* size class; 6 cups total). The *X*. *laevis* were given 24 hours to consume the *Daphnia*, after which any remaining *Daphnia* were counted. There were more replicates of the larval *X*. *laevis* life stage to include a range of developmental stages. Because of the binary results, few replicates were deemed necessary to evaluate the potential for *X*. *laevis* to consume *Daphnia*.

## Results

### Wild *X*. *laevis* Bd infection levels

A total of 70 *X*. *laevis* were collected at the three field sites between November 2012 and May 2015: 31 from the Hedrick Ranch Nature Area (HRNA) pond from four separate visits between November 2012 and May 2014; nine from one visit to Murray Canyon Creek in March 2014; and 30 from five separate visits to Piru Creek pools between May 2014 and 2015. Seven of the *X*. *laevis* were found to be positive for Bd; two from HRNA (2.6; 4.6 Zoospore Equivalents; ZE), one from Murray Canyon (0.32 ZE), and four from Piru Creek (<0.1; 3.4; 3.9; 4.4 ZE) (Table 2). For a complete list of the collection locations, dates, and qPCR results see the Supporting information (S1 Table).

**Table 2:** Bd infection results from live *X*. *laevis* collected from invasive populations in southern California.

Site	Number of *X*. *laevis* Tested	Number Positive	Average Load (ZE)
HRNA	31	2	3.6
Murray Canyon	9	1	0.3
Piru Creek	30	4	2.7

Collection dates ranged between 2012 and 2015. Average loads include infected individuals only.

Four American bullfrogs (*Lithobates catesbeianus*) were also captured at the Piru Creek pools on one site visit and were included in the study. The Piru Creek site is a series of three pools within approximately 200 m of each other where the majority of the *X*. *laevis* were collected from the southernmost pool and the majority of the *L*. *catesbeianus* were collected from the larger northernmost pool. The species co-occurred in the middle pool. The *L*. *catesbeianus* were collected during an invasive species removal project that coincided with visits to this site. All four *L*. *catesbeianus* individuals were Bd positive (1.3; 7.0; 9.6; 421.2 ZE).

### Larval *X*. *laevis* & *Daphnia* consumption of Bd

**Fig 1:**
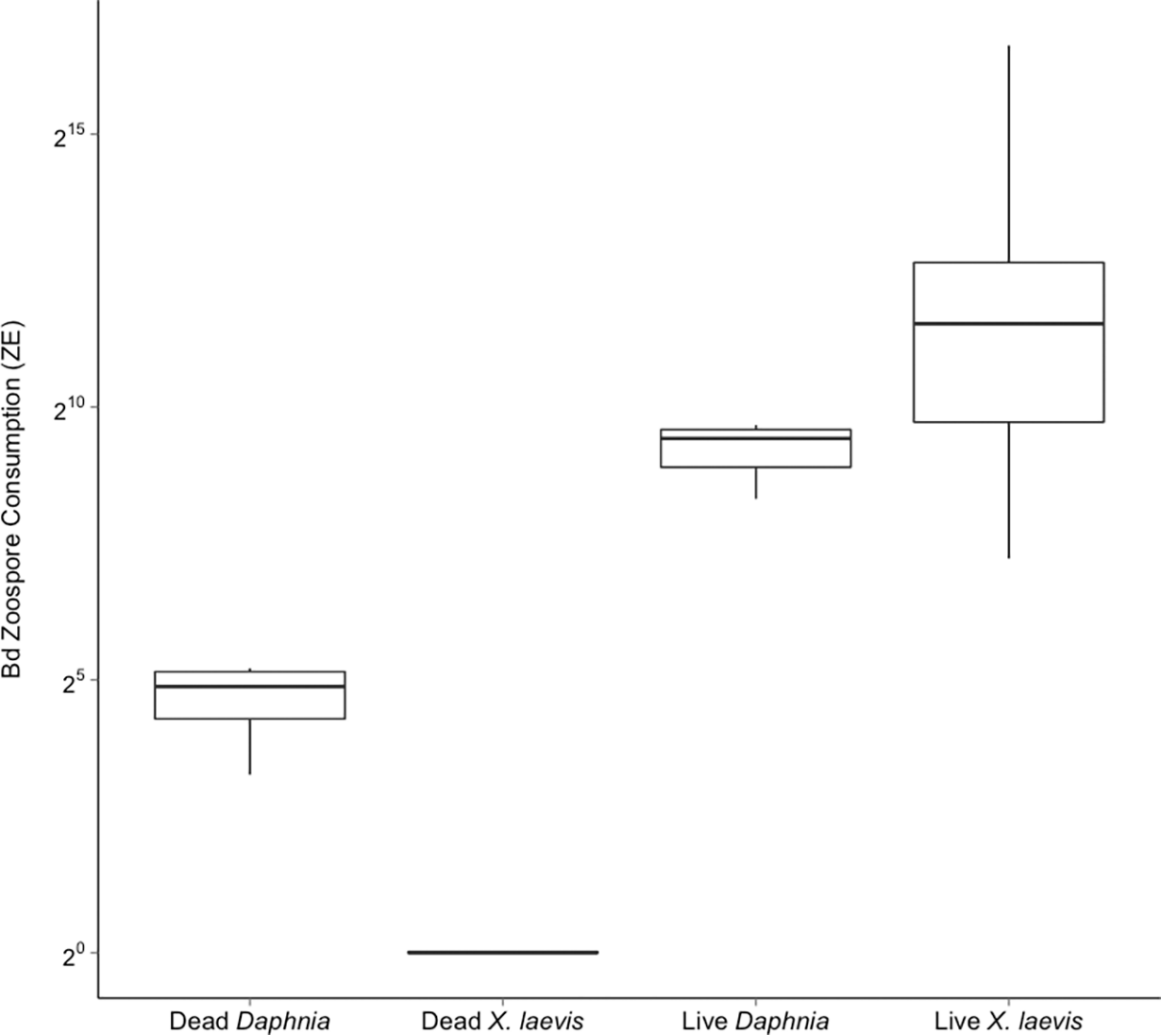
Bd zoospore consumption by *X*. *laevis* and *Daphnia*. Average number of Bd zoospores found in the guts of *X*. *laevis* or on/in the entire three *Daphnia* after 4.5 hours of exposure to 442,000 zoospores. The zoospore values were log transformed to normalize the range of Bd zoospore values. All treatment groups were significantly different from each other (Permutation ANOVA: DF = 3, iterations = 5,000, p = <0.01; FDR p-value adjustments, p<0.05).

Both the larval *X*. *laevis* and the adult *Daphnia* consumed Bd zoospores. A permutation ANOVA analysis was performed on the quantitative PCR results to determine the number of zoospores actively consumed by the *X*. *laevis* and *Daphnia*. The analysis found significant differences between the four treatments tested: live *X*. *laevis*, live *Daphnia*, dead *X*. *laevis*, and dead *Daphnia* (Permutation ANOVA: DF = 3, iterations = 5,000, p = <0.01). Each of the four treatments was significantly different (FDR p-value adjustment, p<0.05) (Fig 1). Larval *X*. *laevis* consumed significantly more Bd zoospores, an average of 11,547 ZE (± 6,545 SE, n = 16), while the sets of three adult *Daphnia* consumed an average of 619 ZE (± 68.2 SE, n = 8), as measured by quantitative PCR of the *X*. *laevis* guts or *Daphnia* bodies. Only trace amount of Bd (0.004 ZE ± 0.001 SE, n = 8) were found in the guts of the dead *X*. *laevis* that were exposed to Bd zoospores. Larger numbers of Bd zoospores were found in the dead *Daphnia* (25.6 ZE ± 6.26 SE, n = 4), likely from zoospores attached to the outer carapace of the crustacean since the entire organism was included in the DNA extraction, rather than only the gut. If the average number of zoospores attached to dead *Daphnia* is subtracted from the set of three *Daphnia*, the average becomes approximately 591 ZE, or 197 ZE for each individual *Daphnia*. Zoospores were largely absent on *X*. *laevis* larvae (n = 5) and *Daphnia* (n = 3) in the negative Bd controls, averaging less than one zoospore for each consumer. The number of zoospores detected in each individual trail is available in the Supporting information (S2 Table).

**Fig 2:**
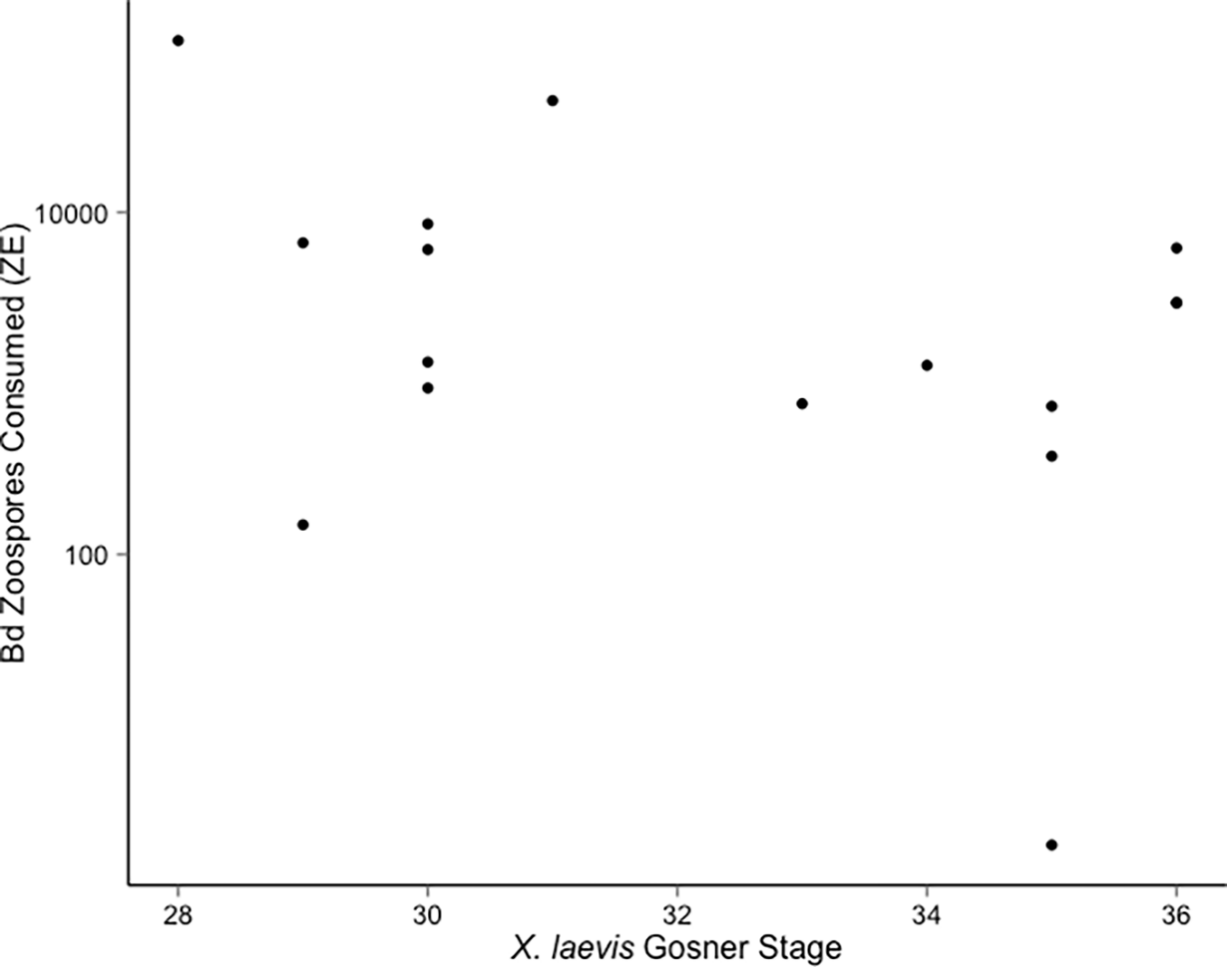
Bd zoospore consumption by *X*. *laevis* developmental stage. Number of zoospores consumed by *X*. *laevis* larvae of varying Gosner developmental stages [32] in the 4.5-hour trials.

There was no significant relationship between the developmental stage of *X*. *laevis* larvae and the number of zoospores an individual consumed (R^2^ = 0.13, F_1,14_ = 2.159, p>0.05) (Fig 2).

### Larval *X*. *laevis* predation on *Daphnia*

Larval *X*. *laevis* (Gosner 26–42) did not consume any *Daphnia* but metamorphic (Gosner 45–46) and juvenile *X*. *laevis* consumed all individuals of all size classes of this crustacean. Two *Daphnia* were found dead but not consumed in treatment cups with larval *X*. *laevis*.

The larval *X*. *laevis* appeared to ignore *Daphnia* while continuously filtering water. The two species were often in close proximity but the *X*. *laevis* were never observed actively moving towards, chasing, or otherwise attempting to capture the *Daphnia*. The *X*. *laevis* metamorphs and juveniles were not observed filter feeding and quickly detect the *Daphnia*. They were observed orienting towards the zooplankter and quickly capture it in a lunging motion.

## Discussion

### Wild *X*. *laevis* Bd infection levels

The results do not support the hypothesis that *X*. *laevis* are reservoirs of Bd in southern California based on the results from the Bd swabs. The three invasive populations of *X*. *laevis* surveyed in this study had a low prevalence of Bd (10%) and a maximum infection load of 4.6 zoospore equivalents (ZE). The combination of low prevalence and low loads suggests it is unlikely that *X*. *laevis* is driving transmission or infection of the pathogen among susceptible amphibians.

The low prevalence and infection load values in this study are comparable to the results from the *X*. *laevis* museum specimens collected across California, 13% Bd prevalence and loads less than one genetic equivalent [17]. However, museum specimens are typically treated with formalin, which degrades DNA. This degradation will make qPCR analysis likely to underestimate Bd presence and loads, particularly on specimens with low infection levels [33]. It is therefore possible that these *X*. *laevis* museum specimens collected in previous decades had higher Bd prevalence and loads. The findings of this study are only slightly higher than histological detection of Bd on museum specimens from California that found 4% of the *X*. *laevis* infected with Bd [18]. The consistently low prevalence from these two previous studies and the live capture specimens from this study suggests that *X*. *laevis* have not been a large reservoir for Bd in California in the past decades nor are they currently.

The prevalence levels in California populations of *X*. *laevis* appear to be higher than France, lower than Chile, and comparable to Japan (Table 1). In their native range, the prevalence of California is lower than some findings but higher than others. However, it is important to consider the methods of Bd detection and quantification as some findings may underestimate Bd prevalence compared to our study. Histology and qPCR based on toe clips may underestimate prevalence or load because the toe reflects only a small portion of the animal, compared to the qPCR swab protocol that wipes of several areas of the frog including the feet. It is unclear why prevalence and loads vary so widely and needs further research.

In our study, animals were collected over several months and years, which may have captured Bd infection variation that appeared to be present in the *X*. *laevis* populations in other regions [19]. Attempts were made to include more populations in this study because the Solís et al. [20] study found Bd positive individuals in only three out of their ten sites but the drought in southern California occurred over the study period and reduced availability of habitats suitable for *X*. *laevis*.

One population of *L*. *catesbeianus* in the series of pools at the Piru Creek site, which co-occurred with some of the *X*. *laevis* included in this study, had higher Bd prevalence and loads than the *X*. *laevis* tested at that site. While this was a small sample size of *L*. *catesbeianus* (n = 4), the high prevalence and loads are consistent with studies from other regions [11, 34, 35]. Despite the higher infection prevalence and loads on the *L*. *catesbeianus* at Piru Creek, the *X*. *laevis* at this site had a 13% infection prevalence and all Bd loads were less than five ZE. This suggests that other amphibians, such as *L*. *catesbeianus*, may be greater reservoir of Bd than *X*. *laevis*.

### Larval *X*. *laevis* & *Daphnia* consumption of Bd

In the laboratory, larval *X*. *laevis* were capable of consuming Bd zoospores from the water column and thereby may act as a biological control agent against Bd. By consuming infectious zoospores, *X*. *laevis* could potentially reduce Bd abundances, leading to lower probability of transmission between amphibians, as has been shown with zooplankton feeding on zoospores in laboratory trials [7, 12, 14, 36]. Larval *X*. *laevis* consumed a large number of Bd zoospores, an average of over 10,000 zoospores per individual in the 4.5 hour trial. The number of Bd zoospores consumed did not vary with *X*. *laevis* larval developmental stage. We expected to observe higher Bd consumption among the largest larvae because of greater size and therefore pumping volumes. It is unclear what caused the lack of a relationship between larval size and Bd consumption. *X*. *laevis* larvae are known to adjust their pumping rate to regulate their ingestion rate at different food particle concentrations [37], so the size of a larvae may not indicate filter rate.

A single *Daphnia* consumed an average of almost 200 zoospores. With these estimates, it would take over 50 *Daphnia* to consume the same number of Bd zoospores as one *X*. *laevis* larva. The *Daphnia* adult and *X*. *laevis* larval consumption rates serve as estimates that do not consider changes in consumption rates over time and under different Bd concentrations. *Daphnia* are discriminant predators and preferentially select prey based on size and structure [38], so the ability to seek out Bd zoospores could make them more efficient predators than the generalist filter feeding *X*. *laevis*, particularly if Bd zoospores are a sought after prey item and present at low concentrations.

### Larval *X*. *laevis* predation on *Daphnia*

Because both larval *X*. *laevis* and adult *Daphnia* consume Bd, intraguild predation could further complicate the potential impacts on Bd. Our results confirm previous work indicating that larval *X*. *laevis* consume small food items such as phytoplankton, and transition to consuming zooplankton during metamorphosis [27]. Despite their large gape, larval *X*. *laevis* did not consume *Daphnia*. Only when the *X*. *laevis* began metamorphosis did they prey upon *Daphnia*. If *X*. *laevis* consume large numbers of *Daphnia*, they could interfere with control of Bd by *Daphnia* or other zooplankton the *X*. *laevis* prey upon.

*Daphnia* are only one of the potential zooplankton predators of Bd. Ciliates and rotifers also consume Bd and research suggests they can reduce the transmission of Bd between amphibians [14]. Rotifers and ciliates have a wide range of sizes and some are less than 200 μm [39], which is within the filtration particle size of larval *X*. *laevis* [24]. The size range of zooplankton could be associated with substantial reduction in zooplankton abundance in the presence of *X*. *laevis*, if larval *X*. *laevis* consume the smaller ciliates and rotifers size classes, while the metamorphosing and metamorphic *X*. *laevis* consume the larger.

### Conclusions

This study suggests that invasive populations of *X*. *laevis* do not act as a major reservoir of Bd infections in southern California. While we found that *X*. *laevis* larvae can consume Bd zoospores, it remains unclear if they could act as a biological control for Bd. Metamorphic *X*. *laevis* consume native zooplankton that may be more effective predators of Bd zoospores. Zooplankton, such as *Daphnia* and ciliates, could reach high enough densities in the environment to reduce Bd zoospore concentrations and Bd transmission. The zooplankton are also more appropriate regulators of Bd abundance than is *X*. *laevis* larvae because they are native to most aquatic systems and have been shown to potentially reduce Bd abundance through predation [13].

Our findings are not an endorsement for the introduction of non-native species to new environments to control Bd. An introduction of *X*. *laevis* could inadvertently bring Bd into the environment since they are carriers of the pathogen and it is unclear how Bd dynamics may change if Bd is already present in the system. Invasive *X*. *laevis* also negatively affect native amphibians and aquatic invertebrates through predation and native amphibian displacement [40, 41, 42], that make them undesirable even if they do not serve as Bd reservoirs.

Our findings stress the importance of exploring different life stages of invasive species to adequately evaluate their impacts to natural systems, particularly for animals such as amphibians, which differ ecologically between larval and adult stages.

## Supporting information

S1 Table qPCR *X*. *laevis* results. (DOCX)

S2 Table Bd zoospore consumption experiment results. (DOCX)
