# Supplementary material for: Invasive African clawed frogs in California: A reservoir for or predator against the chytrid fungus?
Source: PLoS One. 2018 Feb 14;13(2):e0191537. doi: 10.1371/journal.pone.0191537 (PMC5812569; doi:10.1371/journal.pone.0191537)
Supplement: S1 Table — (DOCX) [file pone.0191537.s001.docx]

**S1 Table. qPCR *X. laevis* Results**

Amount of Bd detected on each *X. laevis* collected from three field sites in southern California and dates collected. Each individual *X. laevis* sample was run in triplicate and any positives averaged to create the average Bd load in zoospore equivalents (ZE).

| **Site** | **Sample Date** | **Run 1 Load (ZE)** | **Run 2 Load (ZE)** | **Run 3 Load (ZE)** | **Average Load (ZE)** |
| --- | --- | --- | --- | --- | --- |
| **Hedrick Ranch Nature Area, Ventura County** | 18-Nov-12 | 0 | 0 | 0 | **0** |
|  | 18-Nov-12 | 0 | 0 | 0 | **0** |
|  | 18-Nov-12 | 0 | 0 | 0 | **0** |
|  | 18-Nov-12 | 0 | 0 | 0 | **0** |
|  | 18-Nov-12 | 0 | 0 | 0 | **0** |
|  | 18-Nov-12 | 0 | 0 | 0 | **0** |
|  | 18-Nov-12 | 0 | 0 | 0 | **0** |
|  | 18-Nov-12 | 0 | 0 | 0 | **0** |
|  | 18-Nov-12 | 0 | 0 | 0 | **0** |
|  | 18-Nov-12 | 0 | 0 | 0 | **0** |
|  | 18-Nov-12 | 0 | 0 | 0 | **0** |
|  | 18-Nov-12 | 0 | 0 | 0 | **0** |
|  | 10-Mar-13 | 0 | 0 | 0 | **0** |
|  | 10-Mar-13 | 0 | 0 | 0 | **0** |
|  | 10-Mar-13 | 0 | 0 | 0 | **0** |
|  | 10-Mar-13 | 0 | 0 | 0 | **0** |
|  | 10-Mar-13 | 0 | 0 | 0 | **0** |
|  | 10-Mar-13 | 0 | 0 | 0 | **0** |
|  | 10-Mar-13 | 0 | 0 | 0 | **0** |
|  | 17-Apr-13 | 0 | 0 | 0 | **0** |
|  | 17-Apr-13 | 0 | 0 | 0 | **0** |
|  | 17-Apr-13 | 0.46 | 0 | 8.71 | **4.59** |
|  | 17-Apr-13 | 0 | 0 | 0 | **0** |
|  | 17-Apr-13 | 0 | 0 | 0 | **0** |
|  | 17-Apr-13 | 0 | 0 | 0 | **0** |
|  | 17-Apr-13 | 0.96 | 2.81 | 4.08 | **2.62** |
|  | 11-May-14 | 0 | 0 | 0 | **0** |
|  | 11-May-14 | 0 | 0 | 0 | **0** |
|  | 11-May-14 | 0 | 0 | 0 | **0** |
|  | 11-May-14 | 0 | 0 | 0 | **0** |
|  | 11-May-14 | 0 | 0 | 0 | **0** |
| **Murray Canyon Creek, San Diego County** | 22-Mar-14 | 0 | 0 | 0 | **0** |
|  | 22-Mar-14 | 0 | 0 | 0.32 | **0.32** |
|  | 22-Mar-14 | 0 | 0 | 0 | **0** |
|  | 22-Mar-14 | 0 | 0 | 0 | **0** |
|  | 22-Mar-14 | 0 | 0 | 0 | **0** |
|  | 22-Mar-14 | 0 | 0 | 0 | **0** |
|  | 22-Mar-14 | 0 | 0 | 0 | **0** |
|  | 22-Mar-14 | 0 | 0 | 0 | **0** |
|  | 22-Mar-14 | 0 | 0 | 0 | **0** |
| **Piru Creek, Ventura County** | 6-May-14 | 0 | 0 | 0 | **0** |
|  | 6-May-14 | 0 | 0 | 0 | **0** |
|  | 6-May-14 | 0 | 0 | 0 | **0** |
|  | 6-May-14 | 0 | 0 | 0 | **0** |
|  | 6-May-14 | 0 | 0 | 0 | **0** |
|  | 8-May-14 | 0 | 0 | 0 | **0** |
|  | 8-May-14 | 0 | 0 | 0 | **0** |
|  | 8-May-14 | 0 | 0 | 0 | **0** |
|  | 8-May-14 | 0 | 0 | 0 | **0** |
|  | 8-May-14 | 0 | 0 | 0 | **0** |
|  | 18-Jun-14 | 1.61x10^-4^ | 0 | 0 | **1.61x10^-4^** |
|  | 18-Jun-14 | 0 | 0 | 0 | **0** |
|  | 18-Jun-14 | 0 | 0 | 0 | **0** |
|  | 18-Jun-14 | 0 | 0 | 0 | **0** |
|  | 18-Jun-14 | 0 | 0 | 0 | **0** |
|  | 18-Jun-14 | 0 | 0 | 0 | **0** |
|  | 18-Jun-14 | 0 | 0 | 0 | **0** |
|  | 18-Jun-14 | 0 | 0 | 0 | **0** |
|  | 18-Jun-14 | 0 | 0 | 0 | **0** |
|  | 18-Jun-14 | 0 | 0 | 0 | **0** |
|  | 18-Jun-14 | 0 | 0 | 0 | **0** |
|  | 18-Jun-14 | 0 | 0 | 0 | **0** |
|  | 26-May-15 | 0 | 0 | 0 | **0** |
|  | 26-May-15 | 0 | 0 | 3.90 | **3.90** |
|  | 26-May-15 | 0 | 0 | 0 | **0** |
|  | 26-May-15 | 0 | 0 | 4.36 | **4.36** |
|  | 26-May-15 | 0 | 0 | 0 | **0** |
|  | 26-May-15 | 0 | 0 | 0 | **0** |
|  | 26-May-15 | 0 | 0 | 2.38 | **2.38** |
|  | 26-May-15 | 0 | 0 | 0 | **0** |
