# Supplementary material for: Invasive African clawed frogs in California: A reservoir for or predator against the chytrid fungus?
Source: PLoS One. 2018 Feb 14;13(2):e0191537. doi: 10.1371/journal.pone.0191537 (PMC5812569; doi:10.1371/journal.pone.0191537)
Supplement: S2 Table — (DOCX) [file pone.0191537.s002.docx]

**S2 Table. Bd Zoospore Consumption Experiment Results**

The number of Bd zoospores consumed by each *X. laevis* and group of three adult *D. magna* in the experimental trials. *X. laevis* larvae were measured using three methods: snout-to-vent length (SVL), snout-to-tail length (STL), and Gosner stage. The “Live/Dead” column refers to whether the organism(s) included in that trial were alive or were euthanized beforehand to act as negative controls. The “Bd Zoospore Present/Absent” column refers to whether the water in the trial was spiked with Bd zoospores (present) or acted as a negative control without Bd zoospores (absent). All samples were run in duplicate through qPCR and the zoospore equivalents (ZE) were averaged.

| **Organism** | **Live/Dead** | **Bd Zoospore Present/Absent** | **SVL (mm)** | **STL (mm)** | **Gosner Stage** | **Bd Consumed (qPCR run 1) (ZE)** | **Bd Consumed (qPCR run 2) (ZE)** | **Average Bd Consumed (ZE)** |
| --- | --- | --- | --- | --- | --- | --- | --- | --- |
| *X. laevis* | live | present | 15 | 41 | 36 | 3248.82 | 2642.32 | 2945.57 |
| *X. laevis* | live | present | 14 | 40 | 36 | 7671.94 | 4681.63 | 6176.79 |
| *X. laevis* | live | present | 17 | 52 | 35 | 783.11 | 685.97 | 734.54 |
| *X. laevis* | live | present | 13 | 36 | 35 | 395.37 | 354.18 | 374.78 |
| *X. laevis* | live | present | 13 | 35 | 33 | 814.02 | 708.36 | 761.19 |
| *X. laevis* | live | present | 12 | 37 | 34 | 870.06 | 1677.48 | 1273.77 |
| *X. laevis* | live | present | 13 | 37 | 35 | 0.00 | 3.24 | 1.62 |
| *X. laevis* | live | present | 13 | 37 | 36 | 3340.69 | 2599.98 | 2970.33 |
| *X. laevis* | live | present | 9 | 26 | 29 | 178.70 | 118.57 | 148.63 |
| *X. laevis* | live | present | 9 | 25 | 30 | 5925.42 | 6177.77 | 6051.59 |
| *X. laevis* | live | present | 10 | 26 | 30 | 1263.16 | 1401.34 | 1332.25 |
| *X. laevis* | live | present | 10 | 25 | 28 | 69501.84 | 132251.20 | 100876.52 |
| *X. laevis* | live | present | 11 | 31 | 31 | 47787.28 | 42151.68 | 44969.48 |
| *X. laevis* | live | present | 10 | 27 | 30 | 760.89 | 1117.97 | 939.43 |
| *X. laevis* | live | present | 11 | 28 | 30 | 7250.64 | 9860.00 | 8555.32 |
| *X. laevis* | live | present | 9 | 25 | 29 | 6400.22 | 6865.98 | 6633.10 |
| *X. laevis* | live | absent | 10 | 21 | 31 | 0.01 | 0.00 | 0.01 |
| *X. laevis* | live | absent | 9 | 22 | 31 | 0.01 | 0.00 | 0.01 |
| *X. laevis* | live | absent | 12 | 26 | 34 | 0.00 | 0.00 | 0.00 |
| *X. laevis* | live | absent | 14 | 38 | 35 | 0.01 | 0.00 | 0.01 |
| *X. laevis* | live | absent | 11 | 32 | 34 | 0.05 | 0.02 | 0.04 |
| *X. laevis* | dead | present | 14 | 33 | 34 | 0.01 | 0.01 | 0.01 |
| *X. laevis* | dead | present | 13 | 32 | 35 | 0.01 | 0.00 | 0.01 |
| *X. laevis* | dead | present | 12 | 35 | 35 | 0.01 | 0.01 | 0.01 |
| *X. laevis* | dead | present | 16 | 37 | 35 | 0.00 | 0.00 | 0.00 |
| *X. laevis* | dead | present | 12 | 32 | 29 | 0.00 | 0.01 | 0.01 |
| *X. laevis* | dead | present | 11 | 22 | 28 | 0.00 | 0.00 | 0.00 |
| *X. laevis* | dead | present | 13 | 24 | 29 | 0.00 | 0.00 | 0.00 |
| *X. laevis* | dead | present | 11 | 25 | 30 | 0.00 | 0.00 | 0.00 |
| *D. magna* | live | present | - | - | - | 573.59 | 881.21 | 727.40 |
| *D. magna* | live | present | - | - | - | 829.88 | 761.30 | 795.59 |
| *D. magna* | live | present | - | - | - | 379.97 | 372.53 | 376.25 |
| *D. magna* | live | present | - | - | - | 175.57 | 460.98 | 318.28 |
| *D. magna* | live | present | - | - | - | 959.18 | 668.10 | 813.64 |
| *D. magna* | live | present | - | - | - | 339.68 | 953.16 | 646.42 |
| *D. magna* | live | present | - | - | - | 408.50 | 623.18 | 515.84 |
| *D. magna* | live | present | - | - | - | 761.92 | 752.19 | 757.06 |
| *D. magna* | live | absent | - | - | - | 0.02 | 0.07 | 0.04 |
| *D. magna* | live | absent | - | - | - | 0.00 | 0.00 | 0.00 |
| *D. magna* | live | absent | - | - | - | 0.00 | 0.00 | 0.00 |
| *D. magna* | dead | present | - | - | - | 36.30 | 35.72 | 36.01 |
| *D. magna* | dead | present | - | - | - | 41.14 | 26.79 | 33.97 |
| *D. magna* | dead | present | - | - | - | 19.62 | 27.85 | 23.74 |
| *D. magna* | dead | present | - | - | - | 7.51 | 9.73 | 8.62 |
